# Application of Two Newly Identified and Characterized Feruloyl Esterases from *Streptomyces* sp. in the Enzymatic Production of Ferulic Acid from Agricultural Biomass

**DOI:** 10.1371/journal.pone.0104584

**Published:** 2014-08-05

**Authors:** Misugi Uraji, Jiro Arima, Yoshikazu Inoue, Koichi Harazono, Tadashi Hatanaka

**Affiliations:** 1 Research Institute for Biological Sciences (RIBS), Okayama Prefectural Technology Center for Agriculture, Forestry, and Fisheries, Kaga-gun, Okayama, Japan; 2 Department of Agricultural, Biological, and Environmental Sciences, Faculty of Agriculture, Tottori University, Tottori, Japan; 3 Satake Corporation, Higashi-Hiroshima-shi, Hiroshima, Japan; 4 Enzyme Division, Bio Chemicals Department, Nagase ChemteX Corporation, Fukuchiyama, Kyoto, Japan; University Paris South, France

## Abstract

Ferulic acid (FA), a component of hemicellulose in plant cell walls, is a phenolic acid with several potential applications based on its antioxidant properties. Recent studies have shown that feruloyl esterase (FAE) is a key bacterial enzyme involved in FA production from agricultural biomass. In this study, we screened a library of 43 esterases from *Streptomyces* species and identified two enzymes, R18 and R43, that have FAE activity toward ethyl ferulate. In addition, we characterized their enzyme properties in detail. R18 and R43 showed esterase activity toward other hydroxycinnamic acid esters as well, such as methyl *p*-coumarate, methyl caffeate, and methyl sinapinate. The amino acid sequences of R18 and R43 were neither similar to each other, nor to other FAEs. We found that R18 and R43 individually showed the ability to produce FA from corn bran; however, combination with other *Streptomyces* enzymes, namely xylanase and α-l-arabinofuranosidase, increased FA production from biomass such as corn bran, defatted rice bran, and wheat bran. These results suggest that R18 and R43 are effective FAEs for the enzymatic production of FA from biomass.

## Introduction

Ferulic acid (4-hydroxy-3-methoxycinnamic acid, FA) is a phenolic acid that is found abundantly in the hemicellulose of plant cell walls, where it cross-links arabinoxylan molecules via arabinose residues, in addition to others, within the Poaceae plant family. FA has potential therapeutic applications due to its antioxidant and anti-inflammatory properties [1]. FA moderates oxidative stress and inflammation in Alzheimer's disease [2], [3] as well as reduces DNA damage from irradiation in mammalian cells [4]. FA is also used to produce the flavoring agent vanillin by microbial conversion [5], [6].

Enzymatic production of FA from biomass has been reported previously [7], [8], and feruloyl esterase (FAE) has been identified as a key enzyme in the process [9]. FAE is found in *Aspergillus* species such as *A. niger* [10], *A. awamori* [11], [12], and *A. oryzae* [13]. FAEs are classified into four subgroups, A, B, C, and D, according to their amino acid sequences and substrate specificity [13]. In addition, FAEs from *Streptomyces* species have also been reported [14], [15], however, genetic information on *Streptomyces* FAEs relative to FAE activity is still unclear.

*Streptomyces* is a widely used bacterium and the genomic sequences of several *Streptomyces* species have been identified [16], [17]. Several genes that code for useful enzymes have been identified within the *Streptomyces* genome that are not usually expressed under normal culture conditions. We constructed the enzyme expression system in *Streptomyces* using pTONA vector [18]. This system was able to express *Streptomyces* genes as extracellular proteins.

In this study, we screened 43 esterases from a *Streptomyces* esterase library based on the *Streptomyces* genome. We found two new esterases (i.e., R18 and R43) that had feruloyl esterase activity toward ethyl ferulate. We characterized these enzymes with respect to optimal pH, optimal temperature, and thermal stabilization. Further, we investigated their substrate specificities using ethyl ferulate and methyl-esters of other hydroxycinnamic acids as substrates. In addition, we investigated FA production by R18 and R43 from agricultural biomass such as corn bran, defatted rice bran, and wheat bran.

## Materials and Methods

### Materials

Ethyl ferulate and methyl *p*-coumarate were purchased from Tokyo Kasei (Tokyo, Japan). Methyl ferulate and methyl caffeate were purchased from Santa Cruz (Dallas, Texas, USA). Methyl sinapinate was purchased from Apin Chemicals (Abingdon, Oxon, UK). Methyl vanillate was purchased from Wako (Osaka, Japan). *p*-Nitrophenyl butyrate (*p*NPB) was purchased from Sigma (St. Louis, MO, USA). The *Streptomyces* esterase genes *stx-I* (AB110643) [19] and *stx-IV* (AB110643) [20] were expressed by using the expression vector pTONA5 [18]. Rice bran and corn bran were provided by the Satake Corporation (Higashi-Hiroshima, Japan).

### Sodium dodecyl sulfate-polyacrylamide gel electrophoresis (SDS-PAGE) and N-terminal amino acid sequence analysis of R18 and R43

SDS-PAGE was carried out in 12% (w/v) gel at room temperature (Bio-Rad; Hercules, CA, USA) per the manufacturer's instructions. The gel was stained with GelCode Blue Stain Reagent (Thermo Fisher Scientific; Lafayette, CO, USA). R18 and R43 were transferred onto a polyvinylidene difluoride membrane after SDS-PAGE and loaded onto a protein sequencer (Shimadzu Corp.; Kyoto, Japan) to identify the N-terminal amino acid sequences.

### Enzyme assay

For the assay of FAE activity, ethyl ferulate was used as the substrate. Powdered enzyme R18 or R43 (10 mg) was dissolved in 1 mL water. The protein concentrations of R18 and R43 were 1.73 mg/mL and 1.44 mg/mL, respectively. The reaction mixture consisted of 5 µL enzyme, 4 mM ethyl ferulate, and 50 mM Tris maleate buffer in a total volume of 200 µL. The R18 and R43 mixtures were incubated for 30 min at 50°C and for 30 min at 40°C, respectively. For thermostability measurement, the reaction mixture was incubated at 0–70°C without ethyl ferulate, and FAE activity was measured. The released phenolic compounds were measured by high-performance liquid chromatography (HPLC). One unit of enzyme activity was defined as the amount of enzyme that released 1 µmol of FA per minute. For the assay of the activity of other hydroxycinnamate esters, methyl ferulate, methyl caffeate, methyl *p*-coumarate, methyl sinapinate, and methyl vanillate were used as substrates. The assays were performed using the procedure described above for FAE. A general esterase assay using *p*NPB as substrate was performed, and the released *p*-nitrophenol was quantified by measuring the absorbance at 410 nm.

### HPLC and LC-mass spectrometry (MS) analysis

The components of the reaction mixture were separated using HPLC with a Symmetry C_18_ column (3.5 µm, 2.1×50 mm; Waters; Milford, MA, USA) maintained at 40°C. The separation was performed within 5 min, using a linear gradient of 0.1% formic acid in water containing from 10% to 60% acetonitrile, at a flow rate of 0.3 mL/min. The separated FA, caffeic acid, *p*-coumaric acid, and sinapic acid were detected at 322 nm. The separated vanillic acid was detected at 250 nm. The LC-MS spectra of FA and diferulic acid (di-FA) were detected by electrospray ionization in the positive-ion model (ESI^+^) at an m/z ratio of 195.2 and 385, respectively.

### Enzymatic hydrolysis of biomass

All biomasses were pretreated at 99°C for 5 min. The 800-µL reaction mixture consisted of 10 mg biomass, 5–20 mg powdered enzyme R18 or R43, 5 mg powdered enzymes STX-I and STX-IV, and 50 mM Tris maleate buffer (pH 7.0). After incubating the reaction mixture for 24 h at 40°C with mixing at 1400 rpm, the supernatant was collected after centrifugation. The supernatant was diluted with 0.1% formic acid in water. The released FA was measured by HPLC.

### DNA accession numbers

The accession numbers assigned to the sequences in the DNA Data Bank of Japan (DDBJ) database are as follows: R18, AB921569; R43, AB921570.

### Statistical analysis

The significance of the differences in mean values of FA produced and FAE activity between groups was assessed by the Student's *t*-test. Differences were considered significant at *P*<0.05.

## Results and Discussion

### Screening of FAE activity from the *Streptomyces* esterase library

**Figure 1:**
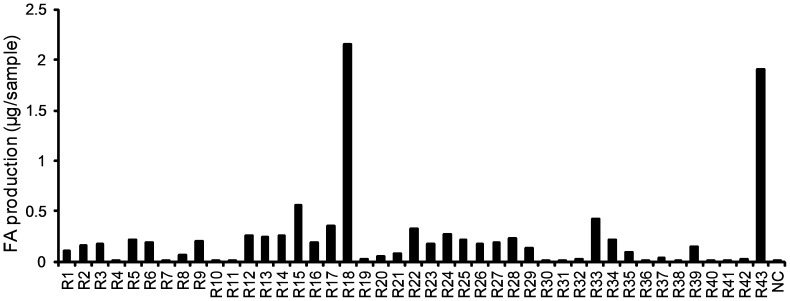
Screening of feruloyl esterases from a *Streptomyces* esterase library.

**Figure 2:**
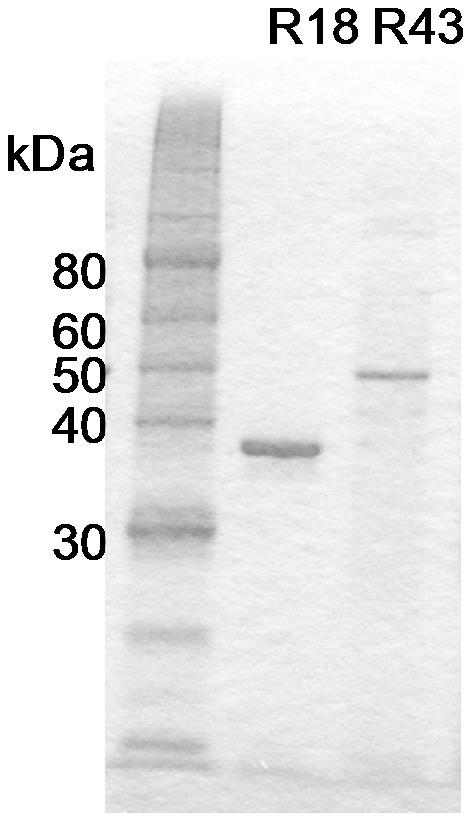
SDS-PAGE analysis of R18 and R43. Lane 1: protein standard; Lane 2: R18; Lane 3: R43. Powdered enzyme (100 µg) was dissolved in distilled water and loaded onto each lane.

The esterases coded by the *Streptomyces* genome were expressed using the *Streptomyces* protein expression system [20]. We screened for enzymes showing FAE activity, using ethyl ferulate as substrate. Almost all the actinomycetes enzymes tested indicated an optimal temperature of approximately 50°C and optimal pH of 6–7 [19], [20], [21], the enzyme reactions were performed at 50°C for 10 h at pH 7. Among the 43 enzymes tested, R18 and R43 indicated high FAE activity (Fig. 1). R18 is a putative esterase from *S. cinnamoneus* and consists of 383 amino acids. A signal sequence was estimated at the N-terminal of the R18 sequence, and the size of the extracellularly expressed enzyme was approximately 38 kDa, which corresponded to the weight of the protein without the signal sequence (Fig. 2). The analysis of the N-terminal sequence of R18 indicated that amino acid residue 42 was the N-terminal of the R18 protein. R43 is another putative esterase from *S. cinnamoneus* and consists of 498 amino acids. The size of the extracellularly expressed enzyme in this case was approximately 52 kDa, which corresponded to the full estimated size of R43 enzyme (Fig. 2). Interestingly, although R43 has no signal peptide for secretion, the enzyme was secreted by the *Streptomyces* protein expression system [18]. The analysis of the N-terminal sequence of R43 indicated that the first amino acid residue was the N-terminal of the R43 protein. Gel filtration results indicated that R18 and R43 had FAE activity as monomers (data not shown). The R18 sequence shared 43.2–46.4% amino acid sequence identity with putative lipases of *S. coelicolor*, *S. lividans, S. clavuligerus* and *S. griseus* (Fig. S1). The R43 sequence shared 42.0–55.8% amino acid sequence identity with putative carboxylesterases of *S. coelicolor*, *S. lividans*, *S. avermitilis* and *S. griseus* (Fig. S2). The amino acid homology between R18 and R43 was very low (20.3%). Although a serine protease motif, “GlyXSerXGly” was identified in R18 and R43 amino acid sequences, other catalytic active site were not clear. In addition, the sequences of R18 and R43 were not assigned to the FAE class of proteins based on their amino acid sequences because they did not share sequence similarity with known FAEs. To clarify the catalytic mechanism of *Streptomyces* FAE and the difference from other FAE, we are attempting the analysis of crystal structure of R18.

### Characterization of R18 and R43 FAE activity

**Figure 3:**
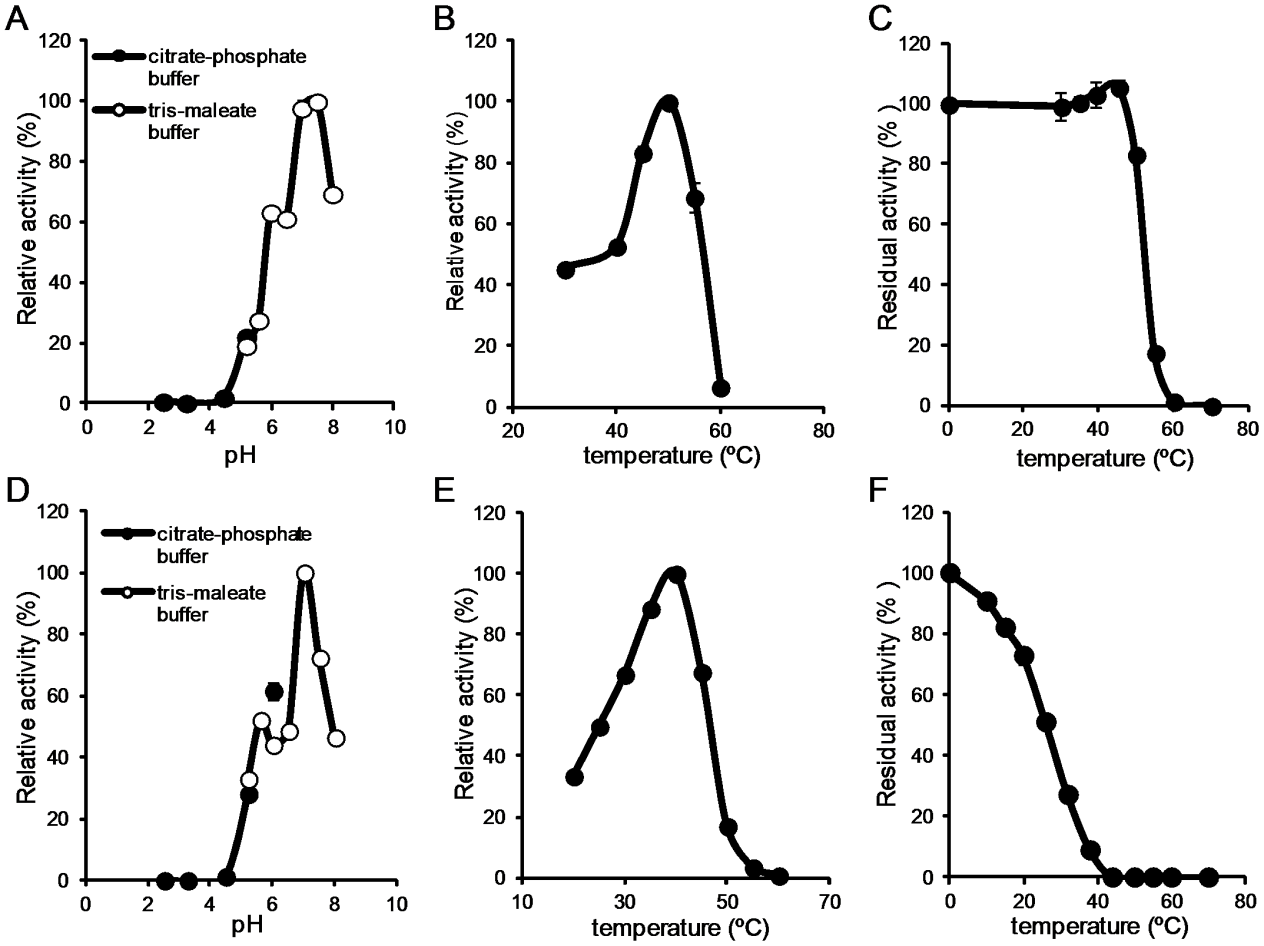
Characterization of the FAE activity of R18 and R43. Effect of temperature (A) and pH (B) on the FAE activity of R18 and thermostability (C). Effect of temperature (D) and pH (E) on the FAE activity of R43 and thermostability (F). Averages from three independent experiments are shown. Error bars represent standard deviations.

We investigated the FAE activity of R18 and R43 at different pH and temperature conditions. The FAE activity of R18 was measured at pH 2.5–8, and the optimal pH was found to be 7.5 (Fig. 3A). The temperature range measured was 30–60°C, and the optimal temperature was 50°C (Fig. 3B). R18 was thermally stable at 45°C and completely inactive at 60°C for 30 min (Fig. 3C).

The FAE activity of R43 was measured at pH 2.5–8, and the optimal pH was 7.0 (Fig. 3D). The temperature range measured was 20–60°C, and the optimal temperature was 40°C (Fig. 3E). R43 was completely inactivated at 40°C for 30 min (Fig. 3F). The FAE activity of both R18 and R43 lasted for 5 h in the presence of ethyl ferulate at 40°C (Fig. S3), suggesting that R43 in the presence of the substrate is stable at 40°C.

### Effect of metal ion and effectors on FAE activity

Next, we evaluated the effect of several metals, ethylenediaminetetraacetic acid (EDTA), ethylene glycol tetraacetic acid (EGTA), and phenylmethylsulfonyl fluoride (PMSF) on the FAE activity of R18 and R43. Among the metals we tested, zinc remarkably reduced the activity of R18 and R43 (Table 1). There were no metal ions capable of activating the FAE activity, whereas EDTA and EGTA did not affect the activity of R18 and R43 (Table 1). PMSF, a serine enzymes inhibitor including serine protease, lipase and esterase, reduced the FAE activity of R18 and R43 to 45.9% and 56.6%, respectively (Table 1). Therefore, we concluded that R18 and R43 belong to the family of serine esterases.

**Table 1:** Effect of metal ion/effectors in R18 and R43.

Metal ion/effectors	Relative activity (%)	
	R18	R43
Control	100±3.1	100±2.4
Na^+^	101.6±1.4	99.4±2.7
K^+^	89.4±2.6	96.2±0.7
Ca^2+^	97.2±5.9	95.9±0.4
Co^2+^	71.4±1.7	87.5±1.5
Fe^3+^	32.6±0.2	83.1±2.4
Mg^2+^	95.0±9.4	99.6±1.5
Mn^2+^	86.0±2.5	95.1±1.6
Zn^2+^	3.9±0.0	3.1±0.1
Ni^2+^	72.3±0.9	88.8±1.1
EDTA	99.0±4.7	101.0±1.8
EGTA	105.5±3.5	97.4±1.8
PMSF	45.9±2.9	56.6±3.8

### Substrate specificity and kinetics of R18 and R43

To evaluate the substrate specificity and kinetics of R18 and R43, ethyl ferulate, methyl ferulate, methyl *p*-coumarate, methyl caffeate, methyl sinapinate, methyl vanillate, and *p*NPB were used as substrates for R18 and R43. Among the five types of hydroxycinnamic acid esters, both R18 and R43 showed their highest activity toward methyl ferulate (23.07 mU/mg for R18 and 19.8 mU/mg for R43), and the *K_m_* values toward methyl ferulate were 4.99 mM and 4.41 mM, respectively (Table 2). Methyl *p*-coumarate, methyl caffeate, and methyl sinapinate were hydrolyzed by R18 and R43, although the esterase activity of both enzymes was lower than their FAE activity (Table 2). The esterase activity of R18 toward all hydroxycinnamic acid esters was higher than that of R43 (Table 2). However, R18 and R43 displayed low esterase activity toward methyl vanillate (1.89 mU/mg for R18 and 0.37 mU/mg for R43), and the corresponding *K_m_* values were not estimated. These results suggest that R18 and R43 prefer cinnamic acid esters as substrates rather than vanillic acid esters. The esterase substrate *p*NPB was tested with both R18 and R43, but only R43 was active against it (0.49 mU/mg, Table 2). The classification of proteins into the classes of FAE is based on their amino acid sequence and substrate specificity [13], [22]. R43 also has broad substrate specificity, similar to R18. These results suggest that R18 and R43 belong to FAEs type C or D.

**Table 2:** Substrate specificity and esterase activity on R18 and R43.

Substrate	R18				R43			
	Km	Vmax	Vmax/Km	Specific activity	Km	Vmax	Vmax/Km	Specific activity
	(mM)	(nmol/min/mg)		(mU/mg)	(mM)	(nmol/min/mg)		(mU/mg)
ethyl ferulate	4.28±0.36	40.64±2.50	9.50	18.97±0.72	1.96±0.14	15.32±0.53	7.83	10.02±0.21
methyl ferulate	4.99±1.72	52.30±9.39	10.48	23.07±0.50	4.41±0.36	41.96±1.93	9.52	19.80±0.72
methyl caffeate	3.31±0.31	7.52±0.63	2.27	5.40±0.18	2.61±0.25	2.10±0.14	0.81	1.54±0.02
methyl *p*-coumarate	4.31±0.26	26.18±0.48	6.07	13.75±0.01	3.00±0.26	8.17±0.63	2.72	6.75±0.12
methyl sinapinate	9.39±1.43	36.70±3.92	3.91	10.90±0.24	0.54±0.07	1.04±0.13	1.93	1.10±0.05
methyl vanillate	-	-	-	1.89±0.03	-	-	-	0.37±0.02
*p*NPB	-	-	-	0.07±0.02	0.17±0.05	0.56±0.02	3.38	0.49±0.00

Average from three independent experiments is shown. Error bars represent standard deviations.

### Release of FA from agricultural biomass by R18 and R43

**Figure 4:**
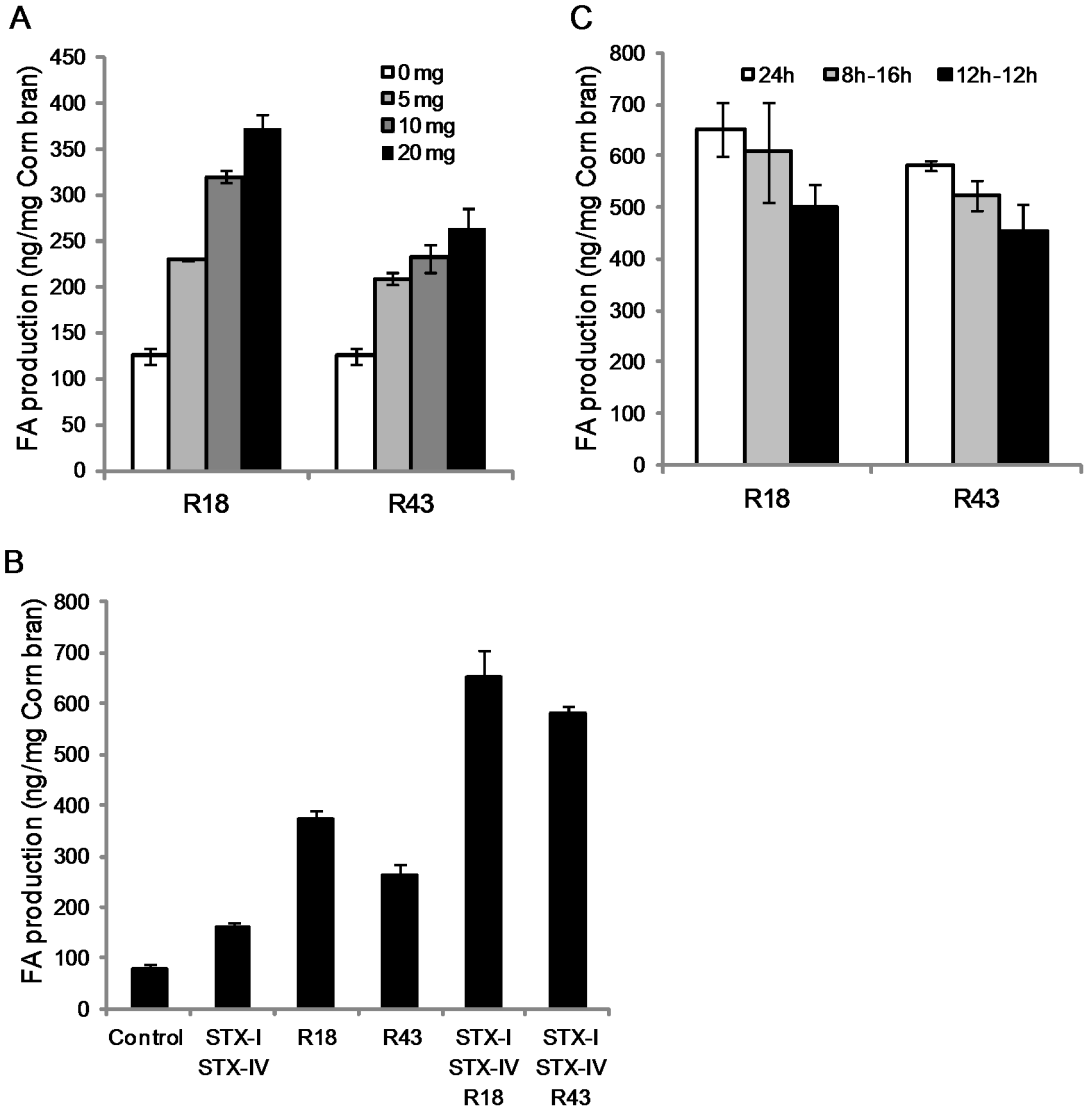
FA production from corn bran by *Streptomyces* FAEs. FA production from corn bran by R18 and R43 (A). Combination effect of xylanase (STX-I) and α-L-arabinofuranosidase (STX-IV) on FA production from corn bran by treatment with R18 and R43 (B). Effect of pretreatment by STX-I and STX-IV on FA production from corn bran by treatment with R18 and R43 (C) The pretreatment of STX-1 and STX-IV was performed during 8 h, 12 h and 16 h. Bars indicate the averages of three independent experiments. Error bars represent standard deviations.

We attempted the production of FA from biomass such as corn bran by treatment with R18 or R43. It has been reported that the combination of xylanase, α-l-arabinofuranosidase, and FAEs leads to increased FA production from biomass [7], [8], [23]. Therefore, we also tested FA production from biomass by using a combination of the xylanase STX-I and the α-L-arabinofuranosidase STX-IV with either R18 or R43. Since R18, R43, STX-I, and STX-IV are active at 40°C and pH 7, these enzymatic reactions were performed at 40°C for 24 h in a buffer at pH 7. When corn bran was treated with R18 or R43 alone, the production of FA increased in a dose-dependent manner (Fig. 4A). The production of FA by treatment with 20 mg R18 enzyme powder was approximately three times higher (372.7 ng/mg of corn bran) than that without enzyme (Fig. 4A). The production of FA by treatment with 20 mg R43 enzyme powder was approximately 2.5 times higher (262.7 ng/mg of corn bran) than that without enzyme (Fig. 4A).

The amount of FA produced by the enzymes combined with STX-I and STX-IV was approximately four times higher (652.8 ng/mg corn bran for R18; 582.4 ng/mg corn bran for R43) than that produced by combining only STX-I and STX-IV (Fig. 4B). These results suggest that STX-I and STX-IV supplied the substrate for R18 and R43 from the biomass. In addition, these results indicate that the FA from biomass increased due to a synergistic effect of STX-I, STX-IV, and either R18 or R43.

Huang et al. [8] reported that pretreatment with xylanase followed by the addition of acetyl xylan esterase (AXE) from *Thermobifida fusca* increased the production of FA from biomass. As shown in Fig. 4C, the amount of FA production after pretreatment with STX-I and STX-IV for 12 h decreased as compared to that after combined treatment with the three enzymes (i.e., R18 or R43, STX-I, and STX-IV) for 24 h. Our results suggest that the mechanism of FA release by R18 and R43 is different from that by AXE.

**Figure 5:**
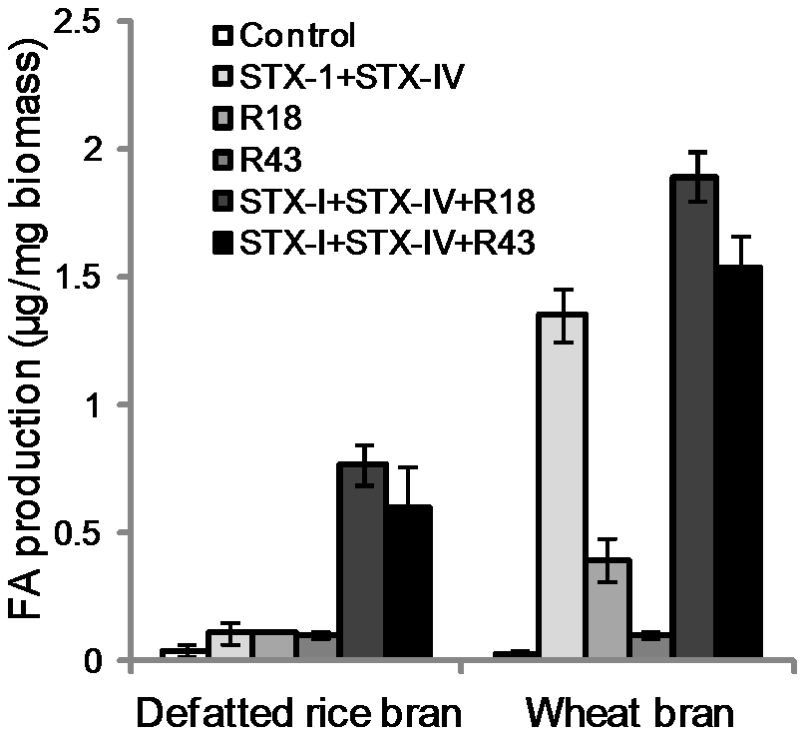
FA production from biomass by *Streptomyces* FAEs. Bars indicate the averages of three independent experiments. Error bars represent standard deviations.

In addition, we tested the production of FA by R18 and R43 from defatted rice bran and wheat bran (Fig. 5). The effect of R18 or R43 single treatment on the production of FA from defatted rice bran was limited. When defatted rice bran was treated with the enzyme combination of STX-I and STX-IV in combination with either R18 or R43, the amount of FA from defatted rice bran increased by up to 6.7 times and 5.8 times, respectively (Fig. 5). The effect of R18 or R43 single treatment on FA production from wheat bran was similar to that of corn bran. In cases of both single and combination treatment, R18 significantly increased FA production from wheat bran as compared to R43 (Fig. 5). The treatment of STX-I and STX-IV was effective on FA production from wheat bran, and the addition of R18 or R43 to this treatment increased FA production (Fig. 5). The plant cell walls are constructed of proteins, starch, fibers and sugars, and the diversity of these compositions has observed among the plant species [24]. Moreover, FA is involved in plant cell walls as sugar modification with various forms [9]. Thus, the effect of *Streptomyces* FAEs might be different on the FA production from different biomass.

**Figure 6:**
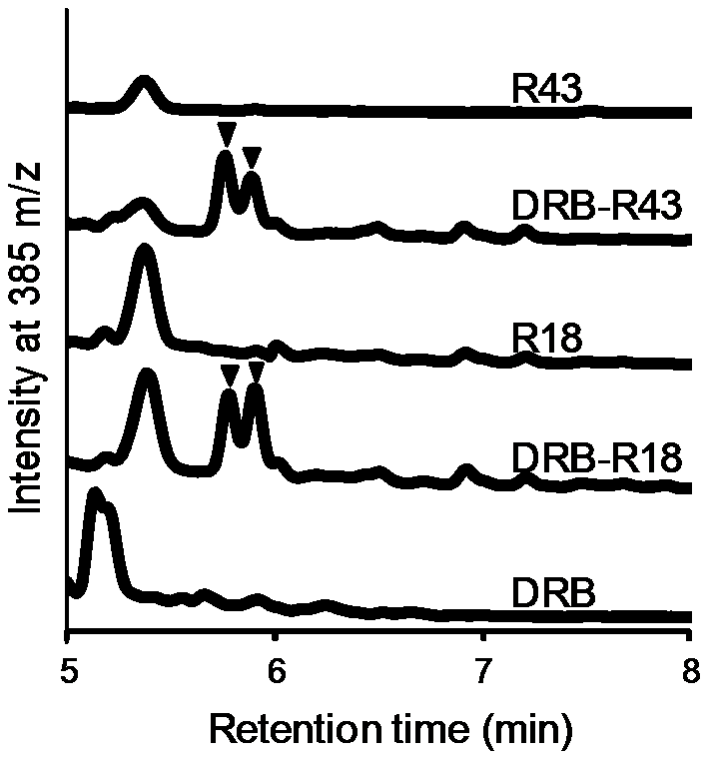
LC-MS plots of defatted rice bran digested by *Streptomyces* FAEs. Arrows indicate estimated di-FAs (m/z = 385).

Several isoforms of di-FA cross-link hemicellulose in the plant cell walls [25], [26]. The release of di-FA is one of the indices for FAE classification [13], [22], [27]. We analyzed the extract from defatted rice bran treated with R18 and R43. The MS signal at m/z 195.2 corresponding to FA was detected in the extract from defatted rice bran treated with the combination of STX-I and STX-IV with R18 or R43, and the retention time was 2.28 min (data not shown). After the elution of FA, two peaks at m/z 385 that were estimated as di-FAs were detected in the extract from defatted rice bran after both R18 and R43 single treatments (Fig. 6) and the enzyme combination of STX-I and STX-IV with R18 or R43 (data not shown). Therefore, we suggest that R18 and R43 belong to type D FAEs.

In contrast to FA, di-FAs were released by R18 and R43, independent of STX-I and STX-IV from defatted rice bran (Fig. 5 and Fig. 6). In addition, the di-FAs released by R18 and R43 from corn bran and wheat bran were undetectable (data not shown). These results suggest that the di-FA released by treatment with R18 and R43 assisted the degradation of hemicellulose of defatted rice bran by xylanase and α-L-arabinofuranosidase. The cooperation of these enzymes might lead to a synergistic effect on FA production.

## Conclusions

R18 and R43, feruloyl esterases from *Streptomyces* sp., were identified by screening a library of *Streptomyces* esterases. Both enzymes belong to type D FAEs based on their substrate specificity and ability to release di-FA. After single treatment with either R18 or R43, FA was released from corn bran and wheat bran. Moreover, the enzyme combination of R18 or R43 with xylanase and α-L-arabinofuranosidase from *Streptomyces* increased FA production from corn bran, defatted rice bran, and wheat bran.

## Supporting Information

Figure S1 **Multiple alignment of R18 and homologues from** ***Streptomcyes*** **sp.** Amino acid sequences were aligned by GENETYX software (Tokyo, Japan) and the amino acid residues matched more than three are indicated in black boxes.
(TIF)

Figure S2 **Multiple alignment of R43 and homologues from** ***Streptomcyes*** **sp.** Amino acid sequences were aligned by GENETYX software (Tokyo, Japan) and the amino acid residues matched more than three are indicated in black boxes.
(TIF)

Figure S3 **Time course of R18 and R43 FAE activity.** Averages of three independent experiments are shown. Error bars represent SD.
(TIF)
