# Supplementary figures and images for: Application of Two Newly Identified and Characterized Feruloyl Esterases from Streptomyces sp. in the Enzymatic Production of Ferulic Acid from Agricultural Biomass
Source: PLoS One. 2014 Aug 5;9(8):e104584. doi: 10.1371/journal.pone.0104584 (PMC4122463; doi:10.1371/journal.pone.0104584)

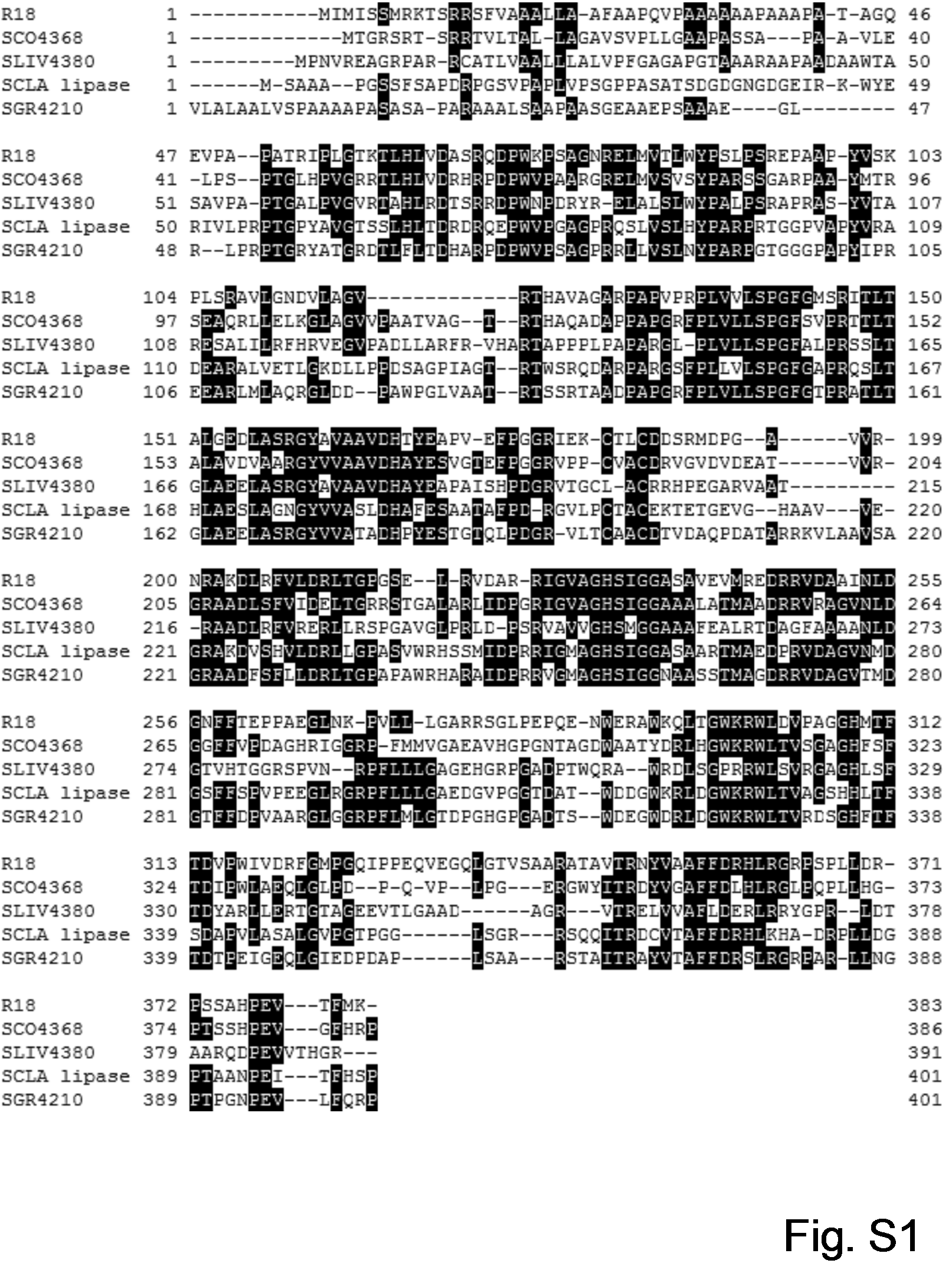

Supplement: Figure S1 — Multiple alignment of R18 and homologues from Streptomcyes sp. Amino acid sequences were aligned by GENETYX software (Tokyo, Japan) and the amino acid residues matched more than three are indicated in black boxes. (TIF) [file pone.0104584.s001.tif]

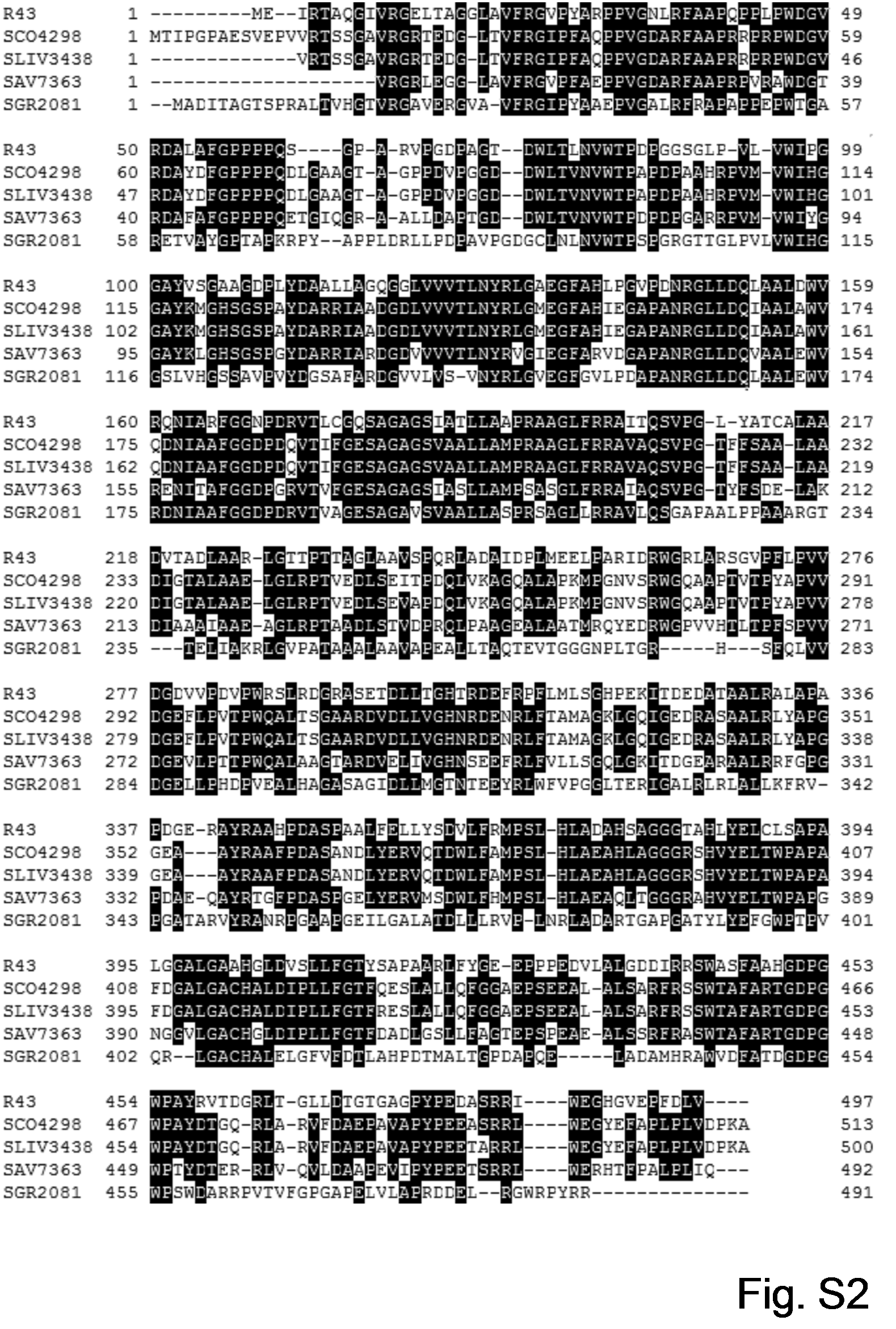

Supplement: Figure S2 — Multiple alignment of R43 and homologues from Streptomcyes sp. Amino acid sequences were aligned by GENETYX software (Tokyo, Japan) and the amino acid residues matched more than three are indicated in black boxes. (TIF) [file pone.0104584.s002.tif]
